# Lymphocyte exhaustion in hepatocellular carcinoma: a dynamic evolution across disease stages

**DOI:** 10.3389/fimmu.2025.1611365

**Published:** 2025-06-06

**Authors:** Carla Fuster-Anglada, Josep Corominas, Aida Marsal, Neus Llarch, Gemma Iserte, Marco Sanduzzi-Zamparelli, Alejandro Forner, Joana Ferrer-Fábrega, Victor Holguin, Albert Morales, Carolina Saavedra, Maria Reig, Loreto Boix, Montserrat Marí, Alba Díaz

**Affiliations:** 1 Pathology Department, Centro de Diagnóstico Biomédico (CDB), Liver Oncology Unit, Hospital Clinic Barcelona, Barcelona, Spain; 2 Barcelona Clinic Liver Cancer (BCLC) Group, Instituto De Investigaciones Biomédicas August Pi i Sunyer (IDIBAPS), Barcelona, Spain; 3 Centro de Investigación Biomédica en Red de Enfermedades Hepáticas y Digestivas (CIBERehd), Madrid, Spain; 4 Facultad de Medicina, Universitat de Barcelona, Barcelona, Spain; 5 Liver Oncology Unit, Liver Unit, ICMDM, Hospital Clinic Barcelona, Barcelona, Spain; 6 Hepatobiliopancreatic Surgery and Liver and Pancreatic Transplantation Unit, Department of Surgery, Liver Oncology Unit, Instituto Clínic de Enfermedades Digestivas y Metabólicas (ICMDM), Hospital Clinic Barcelona, Barcelona, Spain; 7 Department of Molecular and Cellular Biomedicine, Instituto de Investigaciones Biomédicas de Barcelona, Consejo Superior de Investigaciones Científicas (IIBB-CSIC), Institut d'Investigacions Biomèdiques August Pi i Sunyer (IDIBAPS), Barcelona, Catalunya, Spain; 8 Medical Statistics Core Facility, Institut d'Investigacions Biomèdiques August Pi i Sunyer (IDIBAPS), Hospital Clinic Barcelona, Barcelona, Spain

**Keywords:** hepatocellular carcinoma, lymphocytes, NK cells, immune exhaustion, sorafenib

## Abstract

**Background:**

Immune checkpoint inhibitors (ICIs) have transformed cancer therapy. However, their efficacy in hepatocellular carcinoma (HCC) is limited, highlighting the need to further explore immune microenvironments and novel biomarkers. This study examined lymphocyte populations and immune checkpoint dynamics in early, advanced, and post-progression HCC to better understand immune dynamics in HCC and to help identify predictive biomarkers and immune modulation strategies.

**Methods:**

Tumoral and non-tumoral liver tissues were analyzed from HCC patients across early (n=25), advanced (n=22), and advanced-beyond-progression (n=15) stages. Lymphocyte profiling was performed using immunohistochemistry and flow cytometry, focusing on NK cells, T cells, and immune exhaustion markers. An exploratory analysis of this profile and its association with disease progression and recurrence was conducted.

**Results:**

Early HCC exhibited higher liver-resident NK (lrNK) cell densities in non-tumor regions, which diminished with advanced stages. Increased CD56+ cell infiltration in the tumor core was associated with recurrence. Tumor region showed elevated PD-1, NKG2A, and CD39 expression in CD4+ and CD8+ T cells, indicating progressive immune exhaustion. Advanced HCC stages demonstrated altered NK cell phenotypes, with reduced cytotoxic activation (CD16) and increased residency markers (CXCR6/CD69) in tumor-isolated lymphocytes.

**Conclusions:**

Progressive immune exhaustion and dysregulation of lrNK and T cells in HCC reflect the evolution of the immune microenvironment originating in the tumor and leaking into the non-tumoral liver, progressively diminishing the cytotoxic capacity of NK and T cells. CD56+ cell density and immune checkpoint profiles are potential biomarkers for therapeutic response and disease monitoring, underscoring the need for personalized immunotherapy strategies.

## 1 Introduction

The advent of immune checkpoint inhibition therapies has revolutionized cancer treatment over the past decade. These therapies utilize monoclonal antibodies targeting inhibitory checkpoints expressed by immune cells or their ligands expressed by tumor cells. By disrupting these receptor/ligand interactions, the functional inhibition of immune cells can be reversed, restoring effective anti-tumor activity (1). Since the identification of Cytotoxic T-Lymphocyte Antigen 4 (CTLA-4) and Programmed Cell Death Protein 1 (PD-1) as therapeutic targets, the number of recognized checkpoints has grown, and significant efforts are underway to discover new molecules that regulate immune system dynamics (2). Several combinations of immune checkpoint inhibitors (ICIs) and/or anti-angiogenics are in the current list of agents approved by the FDA for the treatment of a wide range of malignancies (3). In hepatocellular carcinoma (HCC) the combinations of atezolizumab/bevacizumab, tremelimumab/durvalumab, nivolumab/ipilimumab, and camrelizumab/rivoceranib are the first-line treatments for unresectable HCC that have been shown to improve survival in phase III trials (4)). However, not all HCC patients are suitable candidates for these combinations and tyrosine kinases scheme is still indicated in more than 25% of patients (5, 6). Therefore, ongoing research into the HCC microenvironment is crucial to enhance understanding of its biology and progression.

Immune checkpoints are not confined to a single cell type and can be expressed in various cell populations depending on physiological conditions or anatomical compartments (7, 8). This is the case for PD-1 and DNAX accessory molecule (DNAM-1), which are expressed in NK and T cell populations respectively (9, 10).

Human circulating NK cells (cNKs) constitute 5-15% of lymphocytes under homeostatic conditions and are classified into CD56^bright^/CD16^neg^ (CD56^bright^) and CD56^dim^/CD16^pos^ (CD56^dim^) NK cells (11). While CD56^dim^ NK cells are abundant in peripheral blood, CD56^bright^ NK cells are the predominant population in the liver. The CD56^dim^ NK cell population predominantly mediates the killing of target cells by secreting perforin and granzymes (12), whereas the CD56^bright^ NK cell population exhibits immunoregulatory and cytokine-producing capacity (13). Recent evidence points to CD56^bright^ and CD56^dim^ NK cells originating from two different progenitor lineages (14).

The liver hosts a unique lymphocyte composition, with NK cells comprising up to 50% of intrahepatic lymphocytes, many of which exhibit tissue-resident characteristics and cannot recirculate (15). Human liver-resident NK (lrNK) cells are distinguished by transcription factors T-bet and Eomes, and surface markers like CXCR6 and CD69 (16, 17). Moreover, lrNK possess more lytic granules, expresses TRAIL and FasL for apoptosis, and produces higher TNF and GM-CSF levels than cNK cells, compensating for reduced pro-inflammatory cytokine production to maintain liver tolerance (18–23). In liver tumors, lrNK cytotoxicity is impaired, as shown by NKG2D downregulation in HCC (24, 25).

Lymphocyte Activation Gene-3 (LAG-3) and T cell Immunoglobulin and Mucin domain-containing-3 (TIM-3) are immune checkpoint markers that suppress effector T cell activity and enhance regulatory T cells (Tregs) (26, 27). LAG-3 inhibits T cells via CD3 crosslinking, while TIM-3 acts as a negative regulator of inflammatory T cells and is expressed on Tregs. Beyond T cells, immune checkpoints also regulate NK cells, balancing activation and inhibition. For instance, NKG2A inhibits NK cells by recognizing HLA, while NKG2D activates them by targeting stress-induced proteins on malignant cells (28). CD16, present in NK^dim^ cells, is a key NK cell activating receptor mediating antibody-dependent cytotoxicity (29). Additionally, DNAM-1 enhances NK cytotoxicity, while T-cell immunoglobulin and ITIM domain (TIGIT) and CD96 inhibit it by competing for shared ligands like CD155 and CD112 (30, 31).

Our study aims to analyze the tumor microenvironment in resected HCC samples via immunohistochemistry and flow cytometry, correlating findings with tumor stage and progression. By profiling lymphocytes in tumoral and non-tumoral liver tissues from patients with varying HCC stages (early, advanced and advanced beyond progression), we sought to uncover immune interactions and identify potential biomarkers of therapeutic response.

## 2 Methods

### 2.1 Patients

This study included 24 early HCC (eHCC) patients who underwent tumor resection, along with one additional patient treated with tumor biopsy and radiofrequency ablation. It also included 22 advanced HCC (aHCC) patients who received percutaneous biopsies prior to initiating systemic treatment with sorafenib, and 15 patients with advanced HCC beyond progression (aHCCbp) who underwent percutaneous biopsies after sorafenib failure and were subsequently treated with nivolumab (anti–PD-1). eHCC patients were defined as BCLC stage 0 or single BCLC A, aHCC patients as BCLC stage B or C, and aHCCbp as BCLC stage B-C beyond progression. Early recurrence was defined as recurrence within one year of HCC resection. All patients were recruited in our center between May 2018 and November 2020. All patients provided written informed consent before enrolment. The study was approved by the Institutional Review Board (HBC/2017/0026 and HCB/2020/0208) and complied with the provisions of the Good Clinical Practice guidelines and the Declaration of Helsinki. Patients’ characteristics are described in **Supplementary Table S1**.

### 2.2 Liver lymphocytes isolation

Liver tissues from liver resection or biopsy were transported at room temperature in complete RPMI 1640 [Sigma, R0883] supplemented with 10% FBS, 1% Pen/Strep [Sigma, P0781-100mL] and 1% L-Gln [Sigma, G7513]. For tumor resected material, 1cm^3^ pieces were cut into smaller fragments and digested in RPMI plus collagenase IV [ThermoFisher, 17104019] 50U/mL for 1 hour at 37°C and constant shaking. Digestion products were mechanically disaggregated using a syringe plunger and filtered through 100µm filters [CellTrics 100µm, Sysmex Partec GmbH ref.04-004-2328]. For biopsy, samples were collected in 1.5mL Eppendorf tubes with 500µL of complete RPMI medium and mechanically disaggregated using RNAse-free disposable pellet pestles [FisherbrandTM Ref.13236679]. The resulting content was then filtered through 100µm filters.

In some cases, lymphocytes were then isolated by density gradient centrifugation using Lymphoprep [Stem Cell Technologies, 07851] according to manufacturer’s instructions.

### 2.3 Flow cytometry

The whole biopsy isolation product, or 500.000 cells from liver resection isolation product, were stained according to previously published protocols (https://www.bdbiosciences.com/en-us/resources/protocols/cell-surface). For intracellular staining, FOXP3 Transcription Factor Staining Buffer Set [eBioscience, 00-5523-00] was used according to manufacturer’s instructions (https://www.thermofisher.com/es/es/home/references/protocols/cell-and-tissue-analysis/protocols/staining-intracellular-antigens-flow-cytometry.html).

The following lymphocyte cells were identified based on combination of surface markers (**Supplementary Table S2**): T cells, T CD4^+^ cells, cytotoxic T CD8^+^ cells, Treg, B cells Natural Killer cells and NK-like CD3^+^ cells. NK cells were further grouped into CD56^+bright^ and CD56^+dim^, which have been reported as granulocytic and cytotoxic, respectively; and into CXCR6^+^CD69^+^ which are associated with liver-residency. The gating strategy used was previously described by our group (32). Briefly, initial gating was performed on single events to exclude cell doublets and aggregates. Lymphocytes were then selected based on their characteristic forward scatter (FSC) and side scatter (SSC) properties. Viable cells were identified and selected using a VivaFix 353/442, (BioRad). T cells were defined by expression of CD3 and further subdivided into CD4^+^ helper T cells, CD8^+^ cytotoxic T cells, and regulatory T cells (Tregs), the latter identified by FOXP3 expression. B cells were identified by CD19 expression, while natural killer (NK) cells were defined by the expression of CD56 and CD16. CD56^+^ cells were defined as lymphocytes expressing the neural cell adhesion molecule (NCAM). This population predominantly includes NK cells (CD3^-^CD56^+^) but may also encompass a population of “NK-like” cells identified based on co-expression of CD3 with CD56 markers. NK and NK-like cells were further categorized into CD56bright and CD56dim subsets. Within each defined immune population, expression of immune checkpoint molecules, including PD-1, DNAM-1, LAG-3, and CD69, was evaluated to assess activation and exhaustion phenotypes. Cell populations labelled with plus or minus signs refer to those cells with (plus) or without (minus) visible expression of the specific markers. In this study, immune exhaustion in T cells was defined by the expression of multiple inhibitory receptors (PD-1, TIM-3, LAG-3, CD39), while for NK cells, exhaustion was inferred based on increased expression of inhibitory receptors (e.g., NKG2A) and reduced expression of cytotoxicity-associated markers such as CD16. Markers like CXCR6 and CD69 were used to define liver-resident NK populations. We acknowledge that NK cell exhaustion remains a debated concept and interpret these profiles as indicative of functional impairment rather than definitive exhaustion.

Mean Fluorescence Intensity (MFI) was computed for all parameters in every analysis and normalized over the Sphero™ Rainbow Calibration Particles (8 peaks), 3.0 - 3.4 µm.

### 2.4 Antibodies and reagents

All antibodies and reagents used are detailed in **Supplementary Table S2** in the **Supplementary Material**. Due to sample limitations, different antibody combinations were used for biopsy-isolated products and liver resection-isolated products (**Supplementary Table S3**). Not all markers could be used in all samples due to cell number limitations. The total sample number analyzed for each marker is detailed in the Results and Figures sections.

### 2.5 Histopathology and immunohistochemistry

The formalin fixed paraffin embedded (FFPE) samples of the 24 eHCC patients were exhaustively reviewed, analysing all the histological features (**Table 1**) from all the tumours for each case, and non-tumoral parenchyma was also reviewed. All the clinical data (**Supplementary Table S1**), as well as follow-up data from all the patients were collected for further correlations with histological and immunohistochemical (IHC) data.

**Table 1:** Histological analysis of surgical resections from eHCC patients according to recurrence detection within the first twelve months post-surgery.

Histological features	All (n= 24)	No recurrence at 12 months (n=16)	Recurrence at 12 months (n=8)	P-value	STD
*WHO grade, n (%)				0.47	
Well differentiated	4 (16.7%)	2 (12.5%)	2 (25.0%)		
Moderately differentiated	16 (66.7%)	12 (75.0%)	4 (50.0%)		
Poorly differentiated	4 (16.7%)	2 (12.5%)	2 (25.0%)		
Size of lesion (mm), median (IQR)	25 (20.5 – 37.0)	23.5 (19.5 – 39.5)	27.0 (23.5 – 33.0)	0.48	0.33
Trabecular pattern, n (%)	19 (79.2%)	11 (68.8%)	8 (100%)	0.08	
Macrotrabecular pattern, n (%)	5 (20.8%)	3 (18.8%)	2 (25.0%)	0.72	0.15
Acinar pattern, n (%)	11 (45.8%)	5 (31.3%)	6 (75.0%)	0.08	0.98
Solid pattern, n (%)	15 (62.5%)	10 (62.5%)	5 (62.5%)	1.00	0.00
Clear cells, n (%)	6 (25.0%)	4 (25.0%)	2 (25.0%)	1.00	0.00
Steatosis lesion, n (%)	6 (25.0%)	5 (31.3%)	1 (12.5%)	0.32	0.47
Steatohepatitis, n (%)	3 (12.5%)	3 (18.8%)	0 (0.0%)	0.19	
Cholestasis, n (%)	3 (12.5%)	2 (12.5%)	1 (12.5%)	1.00	0.00
Satellite lesions, n (%)	2 (8.3%)	1 (6.3%)	1 (12.5%)	0.60	0.22
Vascular invasion, n (%)	7 (29.2%)	5 (31.3%)	2 (25.0%)	0.56	
Tumor necrosis, n (%)	5 (20.8%)	3 (18.8%)	2 (25.0%)	0.72	0.15
Pseudocapsule, n (%)	12 (50%)	7 (43.8%)	5 (62.5%)	0.39	0.38

*WHO, World Health Organization grade of tumoral differentiation.

Tissues suitable for IHC examination were identified in H&E-stained slides, choosing one slide of tumoral and non-tumoral parenchyma of each case, and the paraffin blocks in the archives of our pathology department were accessed. Two sections (4 μm) were prepared on positively charged slides from each paraffin block.

All the antibodies were validated by conventional IHC using tonsil as a control tissue in an automated immunostainer (DAKO; Agilent Autostainer Link 48), and also in which dilution the antibody worked best in case for concentrated antibodies. The process of deparaffinization, rehydration and epitope retrieval were done with PT-Link Rinse Station (Agilent). IHC was performed against CD3 (Polyclonal Rabbit Anti-Human, Ready-to-Use, Agilent), CD4 (Monoclonal Mouse anti-Human, Clone 4B12, Ready-to-Use, Agilent), CD8 (Monoclonal Mouse Anti-Human, Clon C8/144B, Ready-to-Use, DAKO), FOXP3 (1:100 Monoclonal Mouse anti-Human, clone 86D, Anacrom Diagnostics), CD68 (Monoclonal Mouse anti-Human, clone KP1, Ready-to-use, Agilent), CD163 (1:100 Monoclonal Mouse anti-Human, clone 10D6, Biocare Medical), CD38 (1:50, Mouse monoclonal anti-human, clone 38C03, Gennova), CD56 (Monoclonal Mouse anti-Human, clone 123C3, Agilent), CD69 (1:500 Monoclonal Rabbit anti-Human, clone EPR21814, Abcam), CD16 (1:100 Monoclonal Rabbit anti-Human CD16 SP175, Abcam), CXCR6 (1:50 Polyclonal Rabbit anti-Human, Abcam, overnight incubation) and PD-L1 (1:50 Monoclonal Mouse anti-Human, clone 22C3, DAKO). The chromogen used was AEC (3-amino-9-ethylcarbazole, Ready-to-Use, DAKO) and hematoxylin as a counterstain, except for PD-L1 that was performed with hematoxylin-diaminobenzidine (DAB) staining.

### 2.6 Multiplex immunohistochemistry

Multiple immunostains were done using the protocol SIMPLE (sequential immunoperoxidase labelling and erasing) previously described (33) for each of the chosen slides in each case. Positive cell detection in pathology images was analyzed using QuPath software. Initially, it was necessary to validate the effectiveness of the stripping technique eliminating antibodies and preserving antigens using control tissues. Using the SIMPLE protocol, we successfully performed up to four immunohistochemical stains on the same tissue section. Beyond this, tissue quality was compromised.

The different panels were designed to analyse different cell populations in one slide: T lymphocytes (CD3, CD4, CD8, FOXP3 and PD-L1), macrophages (CD68, CD163, CD38 and PD-L1) and NK cells (CD56, CD16, CD69, CXCR6).

### 2.7 Image analysis

After each immunostaining, the slides were scanned before performing the SIMPLE protocol to erase the antibody and start the cycle again. All the slides were digitalized with Ventana DP200 slide scanner (Roche) in 40x magnification. We analysed three spatial compartments for each staining (tumoral, peritumoral and non-tumoral). Manual annotations of the three regions of interest in each histological whole slide image were done. Peritumoral region of interest was 500 mm wide (**Supplementary Figure S1**).

We automatically counted all positively stained cells using the open-source software QuPath (34). The parameters for cell detection and classification were set manually for each staining type, based on the previous experience of Kather et al. (35) and were manually verified to ensure accuracy. We obtained absolute cell density (cell number per mm2). Analysis of twelve stains for 24 patients across the three spatial compartments resulted in 864 cell density points, with 21 missing values due to staining or image quality issues.

### 2.8 Statistical analysis

Results are presented as frequencies and percentages for categorical variables, normally distributed continuous data were summarized as means (SD) or medians and interquartile intervals [IQR: percentile 25th – 75th] for not normal distributions. In univariate statistical comparisons, Chi-squared analysis and Fisher’s exact test was used for categorical variables and continuous variables were compared using parametric t-test and non-parametric Mann-Whitney test. The significance level was set at two-sided 5%. The balance between groups was assessed using standardized mean differences (STD). STD imbalance was defined as an absolute value greater than 0.20 (small effect size). Pearson or Spearman’s rank correlation was used to analyze the correlation between immune cell densities. Statistical analysis was performed using SAS 9.4 software (SAS Institute, Cary, NC, USA).

For flow cytometry data, quantitative variables were expressed as median and interquartile range [IQR 25th-75th percentiles]. Categorical variables were described as absolute frequencies and percentages (%). Comparisons between two groups for quantitative or ordinal variables were assessed by Mann-Whitney U test. Fisher’s exact test was used to compare categorical variables. Paired comparisons were assessed by the signed-rank test for quantitative or ordinal variables, or with McNemar test for categorical variables. All tests were two-sided with p-value < 0.05 considered significant. SAS software, version 9.4, was used for all statistical analyses except for the paired differences of lrNK and their infiltrating counterparts that were calculated and graphically displayed using GraphPad Prism 8.4.1.

## 3 Results

### 3.1 Histological analysis of early HCC patients

We first conducted an exhaustive histological analysis of surgical resections from eHCC. Among the 24 cases analyzed, 16 showed no recurrence within the year of resection, while 8 did. Some of the histological characteristics analyzed are summarized in **Table 1**.

### 3.2 Distribution of immune populations in tumor and non-tumor areas

Given that in eHCC resections the tumor, non-tumor and peritumor areas were clearly discernible (**Supplementary Figure S1**), we next evaluated by IHC if specific types of immune cells were differentially expressed in these regions. In addition, we explored the relation of the findings with early recurrence (**Table 2**). These allowed us to assess the distribution of immune cell populations, obtain cell density data, and provide insights into the immune landscape that might contribute to tumor recurrence and potentially serve as biomarkers for predicting progression.

**Table 2:** Immune cell density populations’ analysis in tumor, peritumor or non-tumor tissue from eHCC patients according to presence of recurrence twelve months post-surgery.

Cell population and area	All (n= 24)	No recurrence at 12 months (n=16)	Recurrence at 12 months (n=8)	P-value*	STD
CD3 tumoral, median (IQR)	279.5 (102.9 – 763.4)	214.7 (102.9 – 1042.6)	292,4 (95.1 – 554.3)	0.93	-0.05
CD3 peritumoral, median (IQR)	789.0 (322.5 – 1896.8)	907.9 (407.1 – 2163.8)	607.6 (304.9 – 1508.6)	0.31	-0.48
CD3 non-tumoral, median (IQR)	214.6 (69.7 – 413.6)	278.2 (133.0 – 426.0)	206.8 (60.0 – 377.9)	0.33	-0.46
CD8 tumoral, median (IQR)	231.7 (51.0 – 408.2)	231.7 (44.8 – 526.6)	235.5 (51.5 – 324.3)	1.00	0.00
CD8 peritumoral, median (IQR)	498.0 (160.7 – 1199.7)	714.9 (226.3 – 1622.7)	359.1 (160.7 – 859.1)	0.52	-0.31
CD8 non-tumoral, median (IQR)	142.8 (0.0 – 455.9)	132.5 (0.0 – 455.9)	156.3 (9.9 – 341.4)	0.97	0.03
CD4 tumoral, median (IQR)	14.0 (8.4 – 43.0)	16.7 (10.3 – 43.0)	12.7 (4.8 – 36.6)	0.52	-0.46
CD4 peritumoral, median (IQR)	27.8 (9.7 – 147.9)	23.4 (9.1 – 171.7)	33.2 (17.8 – 97.3)	0.88	-0.32
CD4 non-tumoral, median (IQR)	12.9 (7.1 – 24.8)	16.5 (7.7 – 41.2)	10.0 (6.8 – 24.8)	0.78	0.13
FOXP3 tumoral, median (IQR)	4.0 (0.8 – 31.9)	7.6 (0.8 – 32.5)	3.2 (1.1 – 8.9)	0.54	-0.28
FOXP3 peritumoral, median (IQR)	28.5 (7.7 – 75.3)	27.2 (1.5 – 193.7)	40.2 (16.2 – 52.8)	0.97	0.03
FOXP3 non-tumoral, median (IQR)	11.3 (1.9 – 25.0)	15.0 (1.7 – 24.6)	11.3 (3.2 – 43.3)	0.82	0.12
CD68 tumoral, median (IQR)	188.8 (81.1 – 600.6)	175.2 (52.5 – 534.9)	227.4 (117.3 – 650.7)	0.41	0.38
CD68 peritumoral, median (IQR)	268.4 (124.9 – 676.0)	237.0 (59.8 – 598.3)	321.9 (185.8 – 676.0)	0.41	0.38
CD68 non-tumoral, median (IQR)	122.0 (88.8 – 198.4)	121.3 (87.7 – 156.9)	213.3 (95.0 – 404.7)	0.15	0.63
CD163 tumoral, median (IQR)	98.2 (13.2 – 605.5)	101.5 (18.2 – 600.2)	94.6 (7.8 – 829.4)	1.00	0.00
CD163 peritumoral, median (IQR)	81.1 (4.2 – 643.9)	59.8 (4.2 – 553.2)	170.4 (5.7 – 933.5)	0.75	0.15
CD163 non-tumoral, median (IQR)	81.0 (5.9 – 200.0)	81.0 (9.7 – 156.2)	104.7 (2.1 – 281.9)	0.74	0.15
CD68CD38 tumoral, median (IQR)	0.0 (0.0 – 0.78)	0.0 (0.0 – 0.8)	0.0 (0.0 – 1.7)	1.00	0.00
CD68CD38 peritumoral, median (IQR)	0.0 (0.0 – 0.35)	0.0 (0.0 – 0.4)	0.0 (0.0 – 4.5)	0.90	0.07
CD68CD38 non-tumoral, median (IQR)	0.0 (0.0 – 0.21)	0.0 (0.0 – 0.0)	8.4 (0.0 – 49.4)	0.03	0.95
CD56 tumoral, median (IQR)	2.6 (0.0 – 14.5)	0.0 (0.0 – 7.7)	28.8 (9.3 – 36.4)	0.01	1.34
CD56 peritumoral, median (IQR)	11.8 (0.0 – 42.1)	12.0 (0.0 – 38.3)	10.5 (4.5 – 43.7)	0.74	0.19
CD56 non-tumoral, median (IQR)	5.5 (1.1 – 14.2)	4.5 (1.0 – 11.8)	9.7 (3.0 – 15.2)	0.37	0.40
FOXP3 tumoral/CD3 tumoral (%), median (IQR)	1.5 (0.3 – 9.6)	1.5 (0.2 – 10.6)	1.2 (0.5 – 6.2)	0.98	0.00
FOXP3 peritumoral/CD3 peritumoral (%), median (IQR)	2.8 (1.4 – 10.1)	2.8 (1.4 – 10.0)	4.2 (1.3 – 21.9)	0.55	0.27
FOXP3 non-tumoral/CD3 non-tumoral (%), median (IQR)	6.2 (1.8 – 12.7)	5.1 (1.1 – 9.9)	5.8 (4.6 – 18.8)	0.22	0.92
CD8 tumoral/CD3 tumoral (%), mean (SD)	74.0 ± 50.7	71.3 ± 58.3	79.4 ± 33.3	0.72	0.17
CD8 peritumoral/CD3 peritumoral (%), median (IQR)	65.2 (52.1 – 96.4)	59.9 (52.1 – 100.2)	68.2 (45.4 – 86.4)	0.38	0.11
CD8 non-tumoral/CD3 non-tumoral (%), median (IQR)	81.2 (0.0 – 218.3)	54.6 (0.0 – 175.2)	104.6 (0.0 – 230.8)	0.50	0.15
CD68CD38 tumoral/CD68 tumoral (%), median (IQR)	0.0 (0.0 – 0.3)	0.0 (0.0 – 0.5)	0.0 (0.0 – 0.3)	0.76	0.00
CD68CD38 peritumoral/CD68 peritumoral (%), median (IQR)	00 (0.0 – 0.2)	0.0 (0.0 – 0.2)	0.0 (0.0 – 0.5)	0.64	0.07
CD68CD38 non-tumoral/CD68 non-tumoral (%), median (IQR)	0.0 (0.0 – 1.2)	0,0 (0.0 – 0.0)	2.3 (0.0 – 14.7)	0.02	0.95
CD68CD38 tumoral/CD163 tumoral (%), median (IQR)	0.0 (0.0 – 0.0)	0.0 (0.0 – 0.0)	0.0 (0.0 – 0.2)	0.82	0.11
CD68CD38 peritumoral/CD163 peritumoral (%), median (IQR)	0.0 (0.0 – 0.0)	0.0 (0.0 – 0.0)	0.0 (0.0 – 0.7)	0.69	0.20
CD68CD38 non-tumoral/CD163 non-tumoral (%), median (IQR)	0.0 (0.0 – 3.0)	0.0 (0.0 – 0.0)	3.0 (0.0 – 21.7)	0.04	0.90
PD-L1 tumoral, median (IQR)	23.8 (3.8 – 77.7)	23.8 (3.8 – 77.7)	26.2 (6.7 – 60.9)	0.87	-0.08
PD-L1 peritumoral, median (IQR)	15.4 (4.9 – 30.7)	15.4 (4.9 – 30.7)	15.3 (3.2 – 40.6)	0.97	0.03
PD-L1 non-tumoral, median (IQR)	2.8 (1.5 – 7.7)	2.3 (1.5 – 5.6)	5.1 (1.9 – 8.8)	0.44	0.33
PDL1% tumoral, median (IQR)	0.5 (0.1 – 1.2)	0.5 (0.1 – 1.6)	0.6 (0.2 – 1.0)	0.95	-0.04
PDL1% peritumoral, median (IQR)	0.3 (0.1 – 0.6)	0.3 (0.1 – 0.6)	0.4 (0.1 – 0.9)	1.00	0.00
PDL1% non-tumoral, median (IQR)	0.1 (0.0 – 0.2)	0.1 (0.0 – 0.1)	0.2 (0.0 – 0.2)	0.60	0.23

*p-value <0,05.

**Figure 1:**
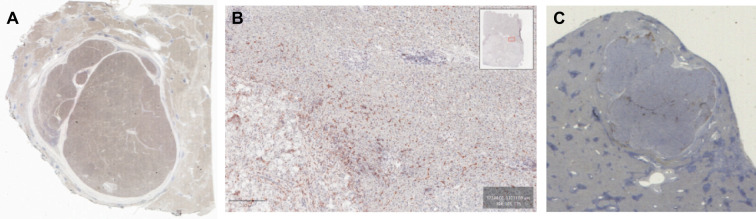
Representative IHC from eHCC **(A)** whole tissue slide displaying CD68 positive staining in tumor, peritumor and non-tumor eHCC; **(B)** inset amplification of CD163 staining in tumor, peritumor and non-tumor eHCC; and **(C)** PD-L1 positive staining in tumoral and peritumoral area.

If we focus on all patients (n=24), some general patterns in the distribution of immune population in tumor and non-tumor areas can be seen (IHC data summarized in **Table 2**). For example, macrophages were present in all compartments (**Table 2**, and CD68+cells or CD163+cells, **Figures 1A, B**). PD-L1 expression was highest in the tumoral area, slightly lower in peritumoral parenchyma, and very low in non-tumoral parenchyma (**Table 2**, **Figure 1C**). We also identified NK cells as CD56+ and were mostly localized in the peritumoral core and non-tumoral parenchyma (**Table 2**).

In a follow-up analysis, we examined which immune population distributions were significantly linked to early recurrence. Our findings revealed that patients with early recurrence had macrophages, identified as CD68+/CD38+ cells, present in the non-tumoral parenchyma (**Table 2**, p<0.03), whereas these cells were absent in patients with no recurrence. Notably, the most important predictor of early recurrence (**Table 2**, p<0.01) was the higher and exclusive presence of CD56+ cells, including NK or NK-like cells, in the tumor area of these patients. To address potential collinearity among immune cell populations, separate logistic regression models were assessed to identify the best predictor of early recurrence. The CD56+ model showed an odds ratio (OR) of 1.11 (95% CI 1.01 – 1.23, p<0.03), suggesting a significant association. The predictive performance of this model, as assessed by the area under the receiver operating characteristic (ROC) curve, was 0.82 (95% CI: 0.60–1.00; p < 0.01), indicating good discrimination for early recurrence.

To further explore the role of NK cells we decided to use flow cytometry. Moreover, to evaluate whether the presence of CD56+ cells in the tumor parenchyma could indicate disease progression, we also included patients at various BCLC-stages in our analysis. This approach enabled us to examine NK cells, along with other lymphocyte populations, to determine if these populations exhibited differential expression and variations in immune exhaustion markers between tumor and non-tumor areas across different BCLC-stages.

### 3.3 DNAM-1, CD96 and TIGIT; NK function regulators, have higher expression on T cells

**Figure 2:**
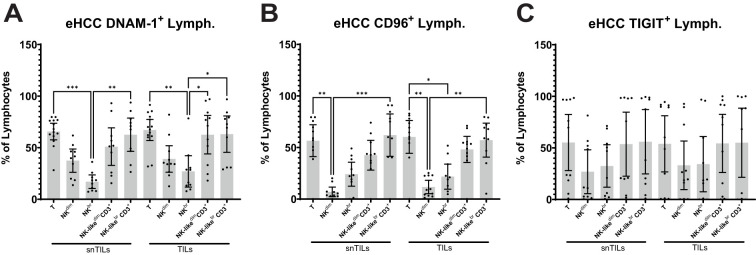
Expression of DNAM-1, CD96 and TIGIT in eHCC patient’s liver-isolated lymphocytes. **(A)** DNAM-1, **(B)** CD96 and **(C)** TIGIT expression was assessed on T and NK TILs and snTILs of eHCC patients (n=14). Lymph, Lymphocytes; eHCC, early HCC patients; snTILs, surrounding non-tumor isolated lymphocytes; TILs, tumor isolated lymphocytes.

We first analyzed the expression of the different CD155 ligands DNAM-1, CD96 and TIGIT, which have shown relevance in the function of NK cells (36). We have evaluated the expression of these three markers in all lymphocytes isolated from eHCC samples. All three markers were found in both T and NK cells, with higher expression in CD3+ cells (either CD56 positive or negative) than in NK cells (either CD56 bright or dim; **Figures 2A-C**) although no statistically significant differences were found for TIGIT. In this study, immune exhaustion in T cells was defined by the expression of multiple inhibitory receptors (PD-1, TIM-3, LAG-3, CD39), while for NK cells, we interpreted phenotypic exhaustion as the co-occurrence of increased inhibitory receptor expression (e.g., PD-1, NKG2A), and decreased cytotoxicity-associated markers (e.g., CD16).

### 3.4 Tumor-isolated lymphocytes exhibit elevated immune exhaustion markers and reduced lrNK cells

**Figure 3:**
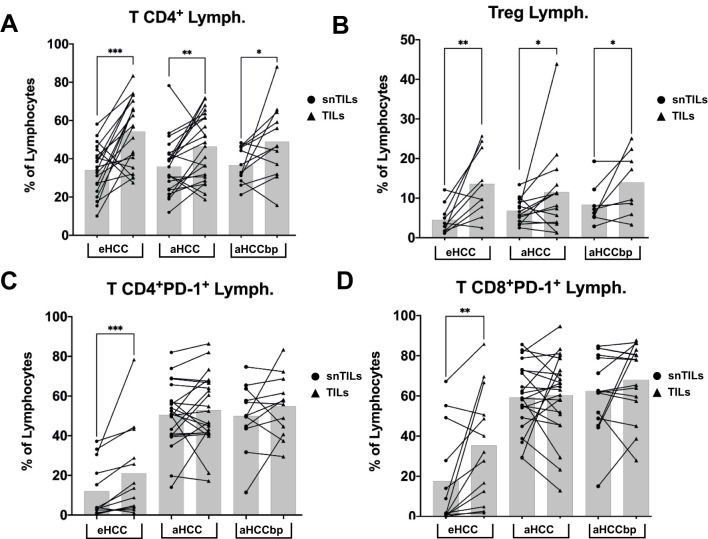
Differences between TILs and snTILs of all groups of patients. TILs from all groups of patients had increased amounts of **(A)** T CD4+ cells and **(B)** regulatory T cells (Treg). PD-1 expression is increased in tumor **(C)** T CD4+ and **(D)** CD8+ cells, compared to non-tumor, in eHCC patients, but not in advanced patients (n = 25). Lymph, Lymphocytes; eHCC, early HCC patients; aHCC, advanced HCC patients; aHCCbp, advanced HCC patients beyond progression; snTILs, surrounding non-tumor isolated lymphocytes; TILs, tumor isolated lymphocytes.

We next compared the phenotype of liver lymphocytes isolated from eHCC patients with aHCC patients, and aHCCbp patients. As shown in **Figure 3**, most differences were observed between tumor isolated lymphocytes (TILs) and surrounding non-tumor isolated lymphocytes (snTILs), with some shared across the three patient groups. Tumor samples from all three groups contained more CD4+ T cells and Tregs than their surrounding non-tumor counterparts (**Figures 3A, B**). Additionally, CD4+ and CD8+ T cells from tumor samples of eHCC patients exhibited higher PD-1 expression compared to their snTIL counterparts (**Figures 3C, D**). These differences were not observed in advanced (aHCC) or advanced beyond progression (aHCCbp) patients, likely due to a general increase in PD-1 expression with advanced stage.

**Figure 4:**
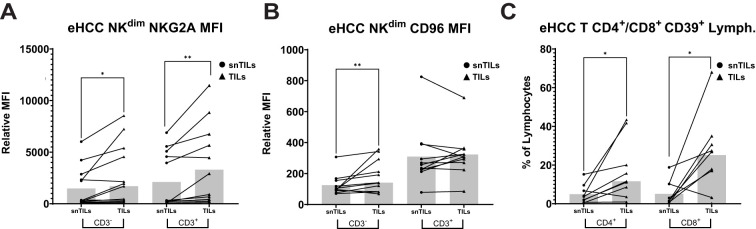
Differences in immune checkpoints expression between TILs and snTILs of eHCC patients. NK^dim^ and NK^dim^-like CD3^+^ cells isolated from tumor samples of eHCC patients had higher expression of **(A)** NKG2A (n=13) and **(B)** CD96 (n=11) compared to their non-tumor counterparts. Both T CD4^+^ and CD8^+^ cells isolated from tumor contained higher amounts of **(C)** CD39^+^ cells (n=9). Lymph, Lymphocytes; eHCC, early HCC patients; MFI, Mean Fluorescence Intesity; snTILs, surrounding non-tumor isolated lymphocytes; TILs, tumor isolated lymphocytes.

Due to the low number of lymphocytes isolated from aHCC and aHCCbp patients’ biopsies, some exhaustion markers were only analyzed in the eHCC group, where more difference could be expected. Among these markers, we observed increased expression of NKG2A in NK^dim^ and NK^dim^-like CD3^+^ cells, as well as increased expression of CD96 in NK^dim^ cells (**Figures 4A, B**). Additionally, CD39 expression, a marker of pathogenic T cells, was elevated in the tumor areas in both CD4+ and CD8+ T cells (**Figure 4C**).

**Figure 5:**
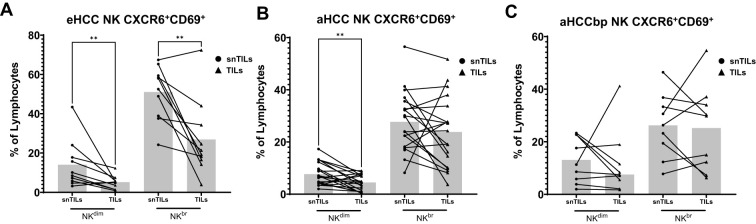
Liver resident NK cells are more abundant in non-tumor tissues and decrease with progression. The presence of lrNK cells was assessed using CXCR6/CD69. **(A)** eHCC (n=11) snTILs exhibit higher numbers of NK^dim^ and NK^br^ liver-resident cells. **(B)** In aHCC patients (n=9), this difference is observed specifically in NK^dim^ cells. However, **(C)** in aHCCbp patients (n=15), no significant differences in lrNK cells are found between snTILs and TILs. Lymph, Lymphocytes; eHCC, early HCC patients; aHCC, advanced HCC patients; aHCCbp, advanced beyond progression HCC patients; snTILs, surrounding non-tumor isolated lymphocytes; TILs, tumor isolated lymphocytes.

When focusing on liver resident NK (lrNK) cells, characterized by the co-expression of CXCR6/CD69, we found that non-tumor areas in eHCC contained a higher percentage of lrNK cells, both CD56^dim^ and CD56^bright^, than in tumor areas (**Figure 5A**). These differences, however, were gradually lost in aHCC patients (**Figures 5B, C**), with aHCCbp patients displaying similar incidence of activated lrNK, CD56^dim^ or CD56^bright^, in tumor and non-tumor areas.

### 3.5 Tumor immune exhaustion expands towards non-tumor surrounding tissues in advanced HCC stages

**Figure 6:**
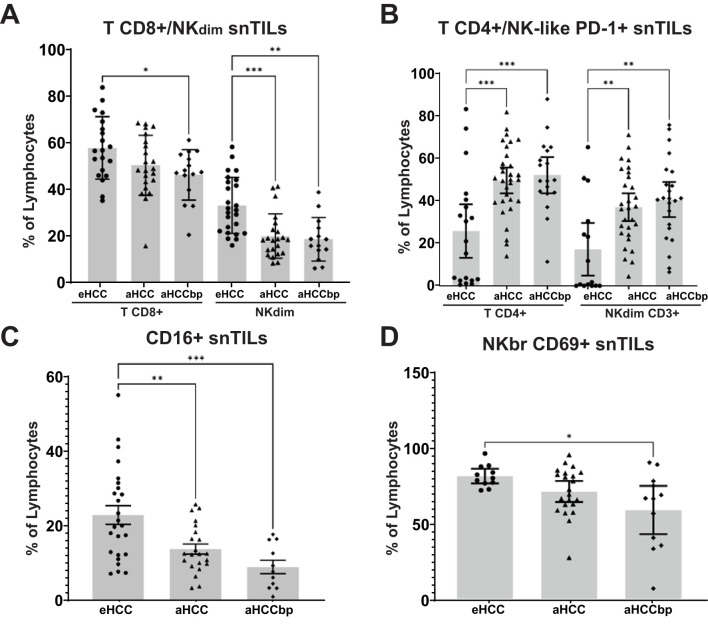
Decrease of cytotoxic lymphocytes and increase of immune exhaustion in snTILs of aHCC patients. **(A)** Percentage of CD8^+^ and NK^dim^ cells isolated from non-tumor tissues of eHCC (n=25), aHCC (n=22) and aHCCbp (n=15) patients. Percentage of **(B)** T CD4^+^ cells and NK-like^dim^ CD3^+^ PD-1^+^ cells, **(C)** CD16^+^ cells and **(D)** NK^br^ CD69^+^ cells. eHCC, early HCC patients; aHCC, advanced HCC patients; aHCCbp, advanced HCC patients beyond progression; snTILs, surrounding non-tumor isolated lymphocytes; TILs, tumor isolated lymphocytes; NK^br^, NK^bright^.

In the surrounding non-tumor tissue, we found that isolated lymphocytes across all three HCC stages revealed a decrease in the number of CD8+ T cells and NK^dim^ cells along HCC progression (**Figure 6A**). These differences were not observed among TILs (**Supplementary Figure S2**). Additionally, we examined the expression of immune checkpoints such as PD-1 in different immune cell populations. Results indicate that T CD4+ and NK-like CD3+ cells from advanced HCC patients’ snTILs exhibit higher PD-1 expression compared to the eHCC group (**Figure 6B**). Furthermore, there was a significative and progressive reduction in the expression of CD16, a marker for NK activation, and CD69, a marker of activation and tissue residency, in NK^bright^ cells (**Figures 6C, D**) with BCLC stage in the surrounding non-tumoral HCC area. These differences, except for PD-1 expression, were not observed among TILs (**Supplementary Figures S2B-D**). All these data confirm that immune exhaustion begins in the tumoral area and expands to the non-tumoral surrounding tissue with progression.

### 3.6 Advanced progression of HCC is related to changes in NK cells of the tumor site

**Figure 7:**
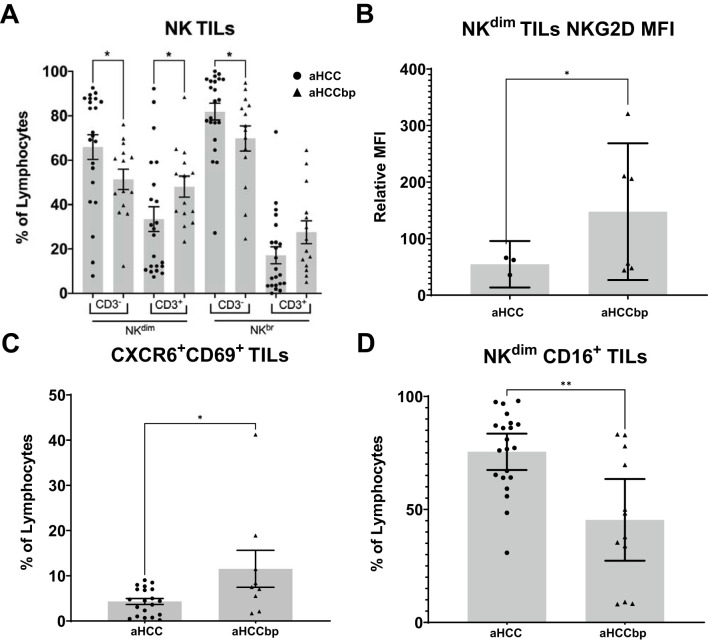
Changes in tumor immune cells of aHCC patients with progression. Tumor NK cells further change their **(A)** CD3^+^/CD3^-^ balance (aHCC/aHCCbp, n=22/15), **(B)** NKG2D expression (n=3/6), **(C)** CD69/CXCR6 expression (n=20/9) and **(D)** CD16 expression (n=21/12) with progression. aHCC, advanced HCC patients; aHCCbp, advanced HCC patients beyond progression; TILs, tumor isolated lymphocytes; MFI, Mean Fluorescence Intensity.

In addition, to assess whether HCC progression affected liver immunity, we next evaluated TILs in the aHCC and aHCCbp groups. In the aHCCbp group TILs showed an increased percentage of NK-like^dim^ and NK-like^bright^ CD3^+^ cells and a reduction in classic NK^dim^ and NK^bright^ CD3^-^ cells (**Figure 7A**). Tumor NK^dim^ cells of the aHCCbp group had higher expression of the activation marker NKG2D and of the markers CXCR6/CD69 than aHCC, associated with liver residency (**Figures 7B, C**) but in contrast had reduced expression of the activation marker CD16 (**Figure 7D**). None of these differences were found when analyzing snTILs (**Supplementary Figures S3A-D**).

**Figure 8:**
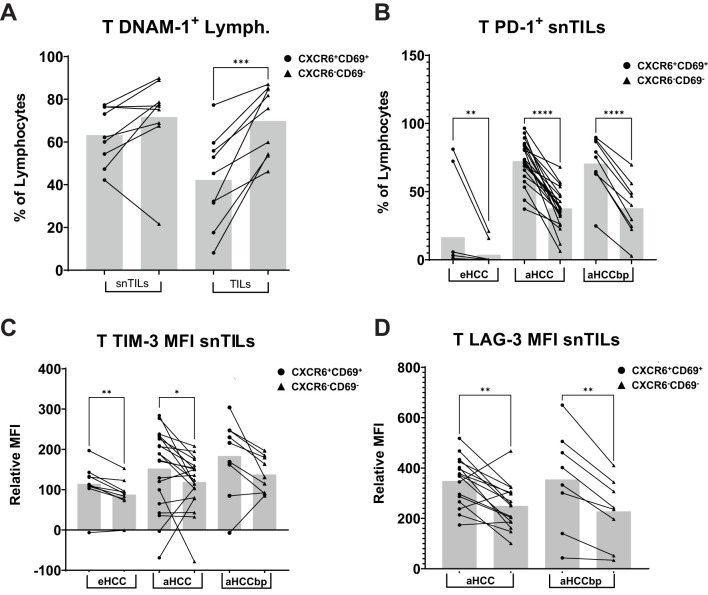
Liver-resident T CXCR6+CD69+ cells have a differential immune checkpoint expression pattern. Liver-resident T CXCR6+CD69+ cells from eHCC patients have lower **(A)** DNAM-1 expression in the tumor area (n=9). In the surrounding non-tumor area, **(B)** PD-1, **(C)** TIM-3, and **(D)** LAG-3 expression is higher in liver resident T cells across all HCC groups (eHCC/aHHC-nt/aHCCbp, n=10/22/9), as compared to non-resident liver T cells, with higher presence of immune exhaustion markers displayed in lymphocytes from advanced patients. Lymph, Lymphocytes; eHCC, early HCC patients; aHCC, advanced HCC patients; aHCCbp, advanced HCC patients beyond progression; snTILs, surrounding non-tumor isolated lymphocytes; TILs, tumor isolated lymphocytes; MFI, Mean Fluorescence Intensity.

Moreover, liver resident T cells also showed lower DNAM-1 expression, an activation marker, in eHCC patients (**Figure 8A**). Consistent with this, further analyses revealed that T CXCR6+CD69+ cells had higher expression of the exhaustion markers PD-1, TIM-3, and LAG-3 in aHCC and aHCCbp groups (**Figures 8B-D**). These differences were also present in tumor-resident T cells (**Supplementary Figures S4A-C**).

## 4 Discussion

This study provides new insights into the evolving immune landscape of HCC, emphasizing the role of NK and T cells in HCC progression and recurrence. While no clear differences in immune exhaustion markers were observed between viral and non-viral cases in our cohort, the potential influence of HCC etiology on immune microenvironment evolution requires further investigation in larger studies. A major strength of our research lies in the stratification of patients into three distinct HCC stages, allowing for a deeper understanding of the evolution of immune dynamics with progression, as well as the direct comparison of lymphocytes from tumor and non-tumor regions at these stages in the same group of patients, which provides a comprehensive view of how immune exhaustion arises and expands during the evolution of the HCC progression. An interesting aspect of our study is the examination of the peritumoral microenvironment through immunohistochemistry, an approach not viable with flow cytometry. Our analysis in early HCC patients reveals that the tumor core and peritumoral parenchyma share similar immunological characteristics, indicating that the tumor influences the immune landscape of the non-tumoral liver from early HCC (eHCC) stages. We observed more significant differences in the non-tumoral parenchyma than in the tumor core between patient groups. This suggests that neoplastic lesions may affect the immune microenvironment throughout the liver. Our findings highlight the presence of immune cell populations linked to a higher risk of eHCC recurrence. Notably, the detection of CD68+/CD38+ macrophages in the non-tumoral tissue is particularly intriguing. CD38 is a molecule involved in the immunosuppressive adenosinergic pathway, which has been linked to T cell suppression (37–39). This could suggest that in the non-tumoral liver of patients with early recurrence, there are signs of an already present immunosuppressive microenvironment that may facilitate tumor recurrence. Moreover, we also detected CD56^+^ NK cell infiltration within tumor cores in patients with HCC recurrence (**Table 2**), emphasizing NK dual role in tumor immunity and immune escape (40), and validating other reports suggesting dysfunctional NK cell activity in HCC (41–44). While NK cell exhaustion remains a debated and evolving concept, particularly in liver immunology, where phenotypic markers often overlap with activation and tissue residency (45, 46), our data provide phenotypic evidence of progressive NK cell dysfunction across HCC stages, characterized by the upregulation of inhibitory receptors and loss of cytotoxic markers, supporting a state of impaired effector potential.

This study has several limitations. First, the sample size, particularly in the advanced HCC (aHCC) and beyond-progression (aHCCbp) groups, was limited due to the difficulty of obtaining longitudinal biopsies in late-stage disease. This reduced the statistical power for subgroup analyses, and while we observed consistent biological trends, not all reached statistical significance. Second, we used a p-value threshold of <0.05 without correcting for multiple comparisons or performing formal power calculations, reflecting the exploratory nature of the study. Another limitation of our IHC study was the inability to fully characterize the activation or inhibition state of CD56+ cells due to technical constraints in staining for CD16, CD69, and CXCR6 markers. While flow cytometry helped to partially address this, spatial resolution of these markers within tissue sections would have provided additional mechanistic insight. Finally, although we profiled immune exhaustion and residency markers in detail, we did not assess functional molecules such as granzyme B or perforin to confirm the cytotoxic potential of NK and CD8+ T cells. Despite these limitations, the observed trends are biologically coherent and support a model of progressive immune exhaustion across HCC stages and tissue compartments.

**Figure 9:**
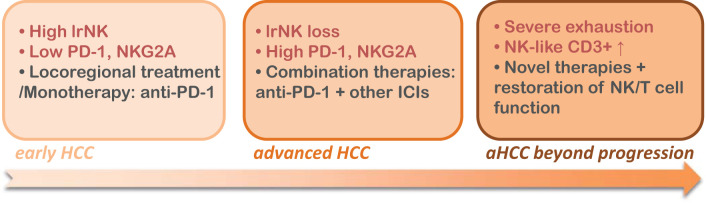
Immune evolution and clinical implications across hepatocellular carcinoma (HCC) progression. Early HCC: Characterized by preserved liver-resident NK (IrNK) cells (CXCR6-CD69+) and low expression of exhaustion markers (PD-1, NKG2A, CD39) in both NK and T cells. This immunologically active profile aligns with curative-intent treatments such as surgical resection or local ablation. If immunotherapy is considered, anti-PD-1 monotherapy may be appropriate at this stage. Advanced HCC (aHCC): Marked by a decline in lrNK cells and increased expression of checkpoint receptors on both NK and T cells, indicating immune dysfunction. Patients typically receive systemic therapies such as tyrosine kinase inhibitors (TKIs) or immune checkpoint inhibitors (ICIs). Combination immunotherapy (e.g., anti-PD-1 plus another ICI, such as anti- NKG2A) may be more effective in addressing emerging exhaustion phenotypes. Advanced HCC beyond progression (aHCCbp): Immune exhaustion is pronounced, with severe reduction in cytotoxic markers and expansion of dysfunctional NK-like cells. These patients are often treatment-refractory and may benefit from clinical trials exploring novel immunotherapeutic strategies and/or strategies to restore NK/T cell function.

Our findings on NK cell dynamics contribute to a deeper understanding of how immune dysfunction impacts NK cells in both tumor and non-tumor areas at various stages of HCC. Notably, DNAM-1, CD96, and TIGIT, as ligands for the CD155 receptor, play pivotal roles in regulating NK cell function. While their role on T cells is less well understood, previous studies have indicated that DNAM-1 expression on T CD4+ cells can mark a suppressive population, whereas its expression on T CD8+ cells is associated with increased cytotoxicity (10). In our analysis in eHCC patients, we found that DNAM-1, CD96, and TIGIT are expressed on both T and NK cells, with unexpectedly higher levels on T cells compared to NK cells. Moreover, the phenotypic changes in NK cells, reduced CD16 expression and increased exhaustion markers such as NKG2A, highlight the impact of chronic tumor exposure on NK cell functionality. Similarly, tumor-infiltrating CD4+ and CD8+ T cells displayed increased PD-1 and CD39 expression, hallmarks of immune exhaustion, particularly in eHCC. These findings confirm that immune dysfunction progressively expands from the tumor to the peritumoral areas and worsens in advanced stages. Of clinical interest, the progressive nature of immune exhaustion, beginning in the tumor and extending into non-tumoral liver tissue, supports a stage-specific approach to immunotherapy. In addition to standard-of-care treatments, immune checkpoint inhibitors may offer benefit across stages: early-stage HCC may be more responsive to single-agent blockade, while advanced stages may require combination strategies to overcome deeper immune dysfunction, as summarized in the **Figure 9**. An initially conflicting result was obtained when observing that NK^dim^ cells within the tumor of aHCCbp patients displayed increased NKG2D expression, as compared to aHCC patients. Still, this was accompanied by a reduction in cytotoxic potential, indicating an ineffective immune response despite apparent activation. While NKG2D is known as an activation marker in HCC (47), and its expression has been observed to be reduced in liver resident NK (lrNK) cells from HCC (24, 25), some reports propose that in advanced cancers driven by inflammation, such as HCC, the expression of NKG2D can drive cancer progression rather than rejection (48, 49)). In this regard, our data supports the involvement of NKG2D in promoting advanced progression. It would be interesting, from a mechanistic point of view, to further assess whether long-lasting NKG2D engagement drives NK cell anergy therefore suppressing their cytotoxic function. In fact, soluble NKG2D ligands, such as sMICA, increased in HCC, can act as decoy receptors stabilizing and blocking NKG2D expression in advanced HCC (50). Regarding MICA, an association between MICA polymorphisms with HCC risk has been described for patients with HCV, suggesting that genetic variants of MICA are of relevance by impacting host immune response (51). Although we did not perform functional assays, future studies evaluating NK cell cytotoxicity and cytokine production will be essential to confirm whether the observed phenotypic exhaustion corresponds to functional impairment in the context of HCC. Further research in this area will be essential to fully understand the role of NKG2D and its ligands in advanced HCC. In this sense, emerging spatial multi-omics approaches, such as high-plex protein and whole transcriptome co-mapping at cellular resolution with spatial CITE-seq, or multimodal tri-omics mapping (52, 53), offer powerful tools for future studies aiming to unravel the spatial and functional heterogeneity of immune exhaustion and tumor-immune interactions in HCC.

Our findings illustrate that lrNK cells, characterized by CXCR6 and CD69 expression, are more abundant in non-tumor areas during eHCC but decline with disease progression, consistent with previous reports (24). The loss of lrNK cells in advanced stages in non-tumor areas suggests impaired hepatic immune surveillance, which could facilitate tumor growth. Altogether, our profiling of NK cell dynamics in HCC is consistent with recent studies characterizing the rapid loss of effector functions and change of phenotype of NK cells within the tumor (54). In addition, our study of T cell dynamics (**Figure 8**) shows that immune exhaustion and inactivation is greater in liver-resident T cells, compared to non-resident, with an increasing exhaustion profile with progression, both in the tumor and non-tumor area.

We also studied NK-like or CD3+ NK cells, in addition to conventional NK cells. In eHCC, the presence of immune exhaustion markers such as CD96 or NKG2A on NK-like cells is more prevalent than on NK cells. Furthermore, within the tumor, the presence of NK^dim^ cells decreases with progression together with an increase in NK-like cells. Interestingly, again, the number of PD1+ NK^dim^-like cells also increase with progression, stressing once again that immune exhaustion is an evolving condition and that the inability to fight the tumor comes from the inside out.

Our comparison of immune cells in the tumor versus surrounding tissues reveals that immune suppression is evident in tumor tissues from the early stages of HCC. Importantly, our study includes lymphocytes from the same patients with HCC at different stages and not from healthy subjects. We are confident that this is a strength of our data as it truly represents the actual immune status of these livers and validates the use of angiotensin-converting enzyme inhibitors at these stages. Indeed, the suppression we observed is manifested both in functional alterations and in changes in the balance of the immune cell population. Further analysis of the surrounding non-tumor tissues from the three patient groups revealed significant differences in both the balance of the lymphocyte population and in markers of immune exhaustion. In aHCC patients, there was a notable reduction in the number of CD8+ T cells and NK^dim^ cells, both of which are crucial for cytotoxic activity. Overall, our data underscores the complexity of immune modulation in HCC and its potential implications for therapy. The strong association between immune checkpoint expression and HCC progression validates the notion that combinatorial strategies targeting multiple checkpoints are mandatory in aHCC. Furthermore, the identification of CD56^+^ cell density and immune exhaustion markers as potential prognostic biomarkers provide a basis for personalized immunotherapy approaches. Further studies are warranted to validate these findings and explore the therapeutic potential of restoring NK or NK-like cell functionality combined with reversing immune exhaustion.

## Data Availability

The original contributions presented in the study are included in the article/**Supplementary Material**. Further inquiries can be directed to the corresponding authors.
