# Correction: Lymphocyte exhaustion in hepatocellular carcinoma: a dynamic evolution across disease stages

**DOI:** 10.3389/fimmu.2026.1799354

**Published:** 2026-02-05

**Authors:** Carla Fuster-Anglada, Josep Corominas, Aida Marsal, Neus Llarch, Gemma Iserte, Marco Sanduzzi-Zamparelli, Alejandro Forner, Joana Ferrer-Fábrega, Victor Holguin, Albert Morales, Carolina Saavedra, Maria Reig, Loreto Boix, Montserrat Marí, Alba Díaz

**Affiliations:** 1 Pathology Department, Centro de Diagnóstico Biomédico (CDB), Liver Oncology Unit, Hospital Clinic Barcelona, Barcelona, Spain; 2 Barcelona Clinic Liver Cancer (BCLC) Group, Instituto De Investigaciones Biomédicas August Pi i Sunyer (IDIBAPS), Barcelona, Spain; 3 Centro de Investigación Biomédica en Red de Enfermedades Hepáticas y Digestivas (CIBERehd), Madrid, Spain; 4 Facultad de Medicina, Universitat de Barcelona, Barcelona, Spain; 5 Liver Oncology Unit, Liver Unit, ICMDM, Hospital Clinic Barcelona, Barcelona, Spain; 6 Hepatobiliopancreatic Surgery and Liver and Pancreatic Transplantation Unit, Department of Surgery, Liver Oncology Unit, Instituto Clínic de Enfermedades Digestivas y Metabólicas (ICMDM), Hospital Clinic Barcelona, Barcelona, Spain; 7 Department of Molecular and Cellular Biomedicine, Instituto de Investigaciones Biomédicas de Barcelona, Consejo Superior de Investigaciones Científicas (IIBB-CSIC), Institut d’Investigacions Biomèdiques August Pi i Sunyer (IDIBAPS), Barcelona, Catalunya, Spain; 8 Medical Statistics Core Facility, Institut d’Investigacions Biomèdiques August Pi i Sunyer (IDIBAPS), Hospital Clinic Barcelona, Barcelona, Spain

**Keywords:** hepatocellular carcinoma, lymphocytes, NK cells, immune exhaustion, sorafenib

Affiliation “4. Facultad de Medicina, Universitat de Barcelona, Barcelona, Spain” was omitted for author “Carla Fuster-Anglada”. This affiliation has now been added for author “Carla Fuster-Anglada”.

The original version of this article has been updated.

