# Supplementary figures and images for: Lymphocyte exhaustion in hepatocellular carcinoma: a dynamic evolution across disease stages
Source: Front Immunol. 2025 Jun 6;16:1611365. doi: 10.3389/fimmu.2025.1611365 (PMC12179174; doi:10.3389/fimmu.2025.1611365)

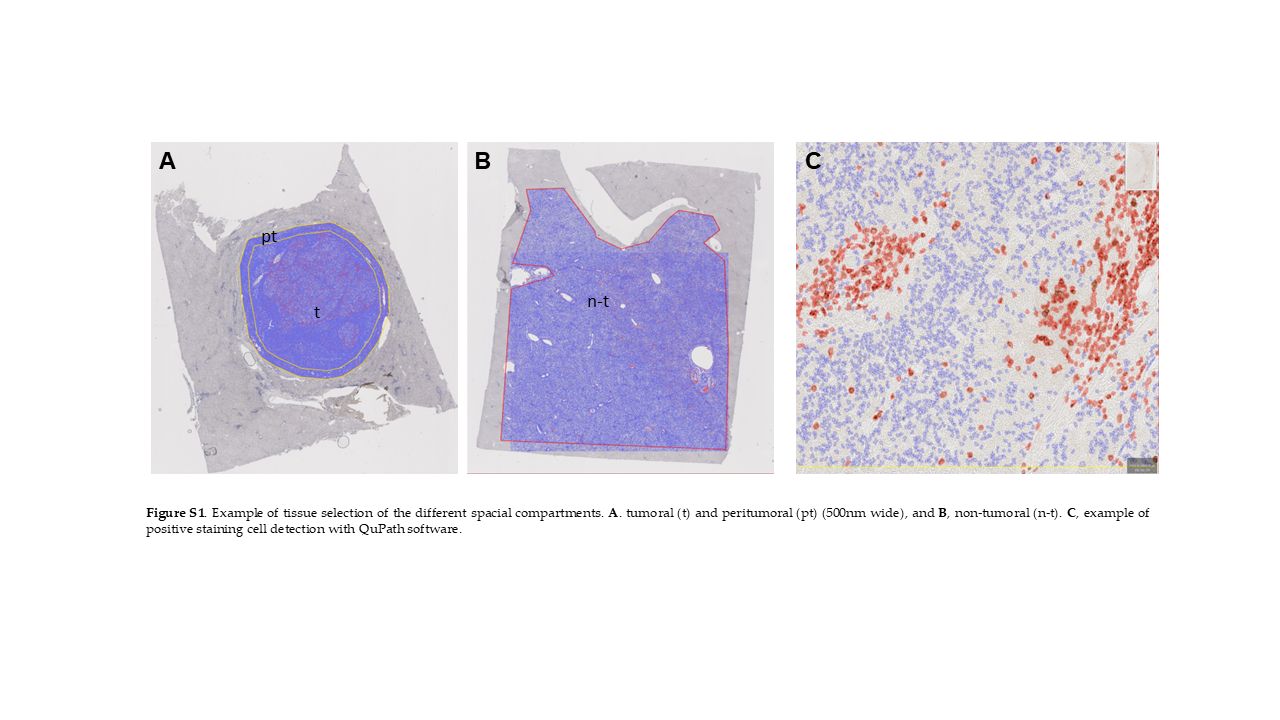

Supplement: Supplementary file 1 [file Image1.tif]

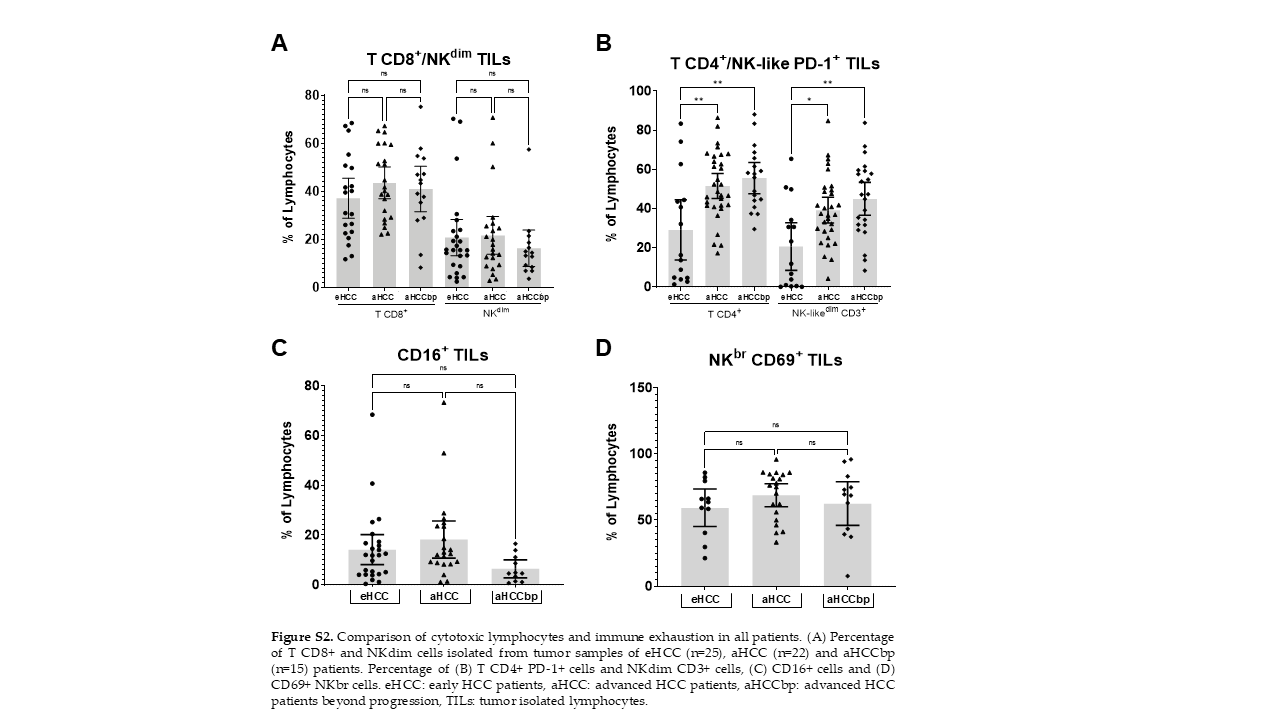

Supplement: Supplementary file 2 [file Image2.tif]

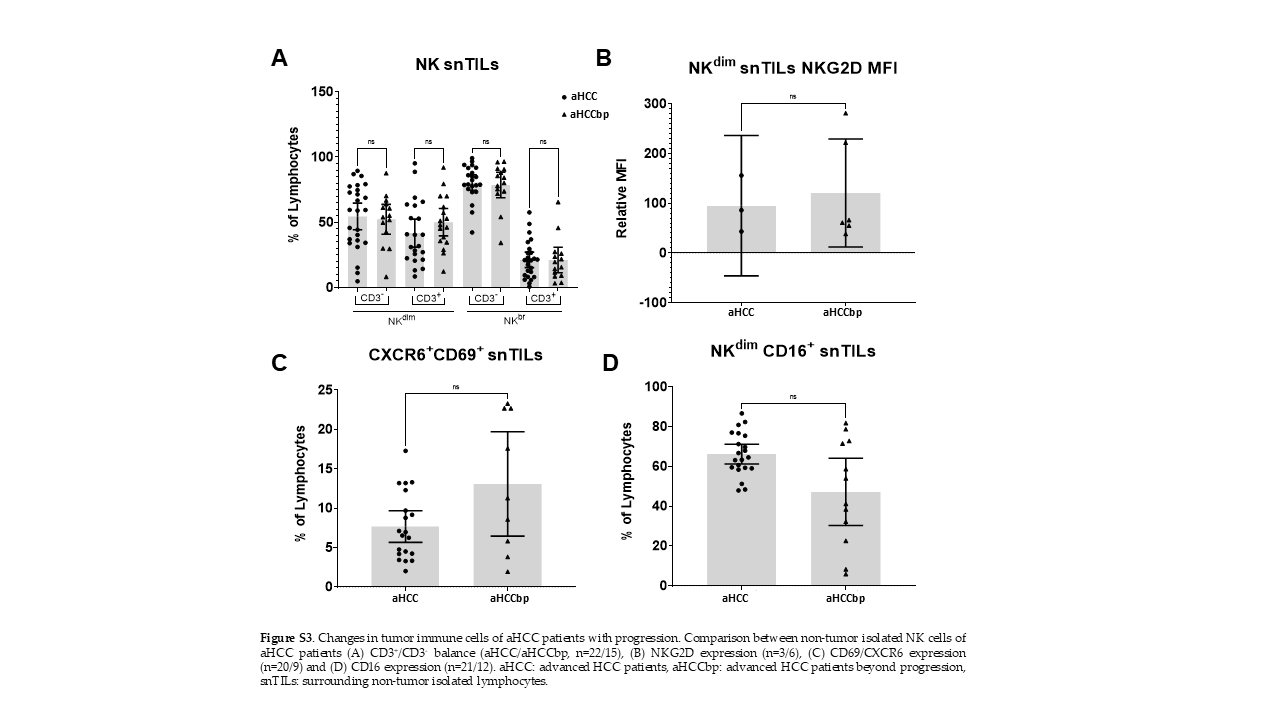

Supplement: Supplementary file 3 [file Image3.tif]

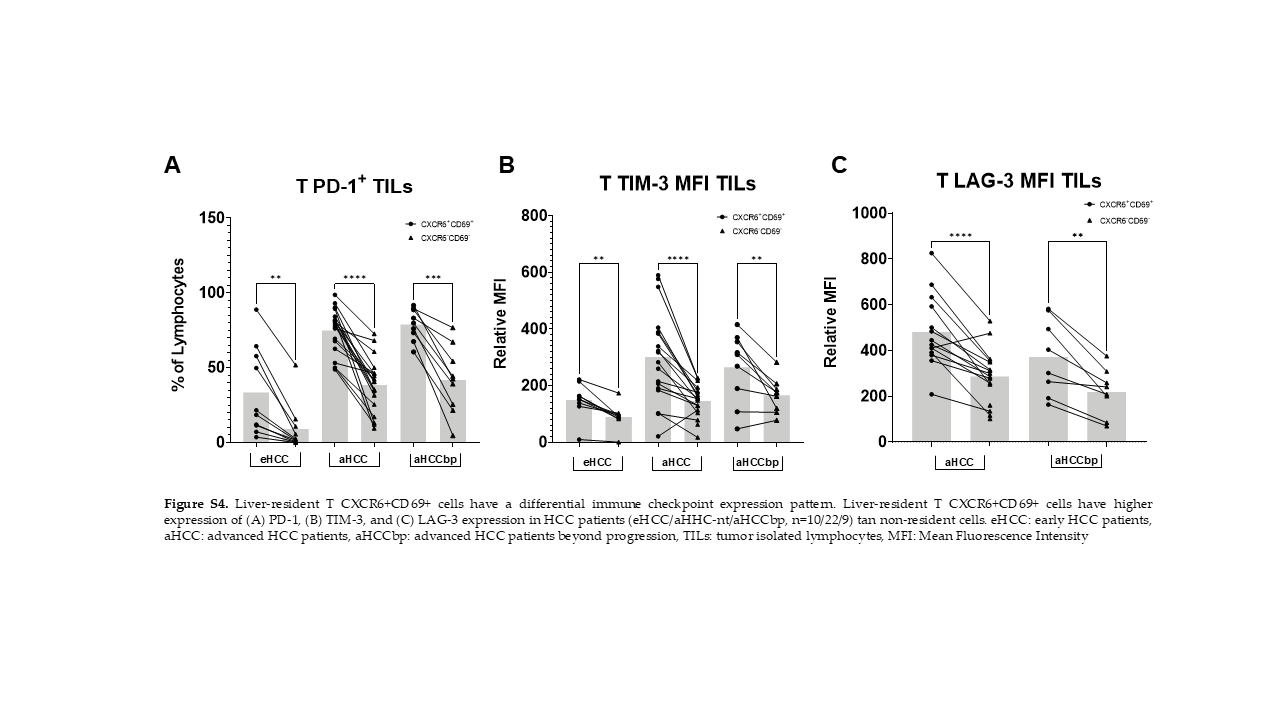

Supplement: Supplementary file 4 [file Image4.tif]
