# Supplementary material for: Lymphocyte exhaustion in hepatocellular carcinoma: a dynamic evolution across disease stages
Source: Front Immunol. 2025 Jun 6;16:1611365. doi: 10.3389/fimmu.2025.1611365 (PMC12179174; doi:10.3389/fimmu.2025.1611365)
Supplement: Supplementary file 5 [file Table1.docx]

Supplementary Material

**Table S1.** Characteristics of the patients with eHCC included in the study

|  |  |  |  |  |  |
| --- | --- | --- | --- | --- | --- |
| Patient's Characteristics | All (n= 24) | No recurrence at 12 months (n=16) | Recurrence at 12 months (n=8) | P-value | STD |
| Age (years), mean (SD) | 64.3 ±10.3 | 64.8 ±9.5 | 63.4 ±12.6 | 0.76 | -0.13 |
| Gender (Male), n (%) | 20 (83.3%) | 13 (81.3%) | 7 (87.5%) | 0.70 | 0.17 |
| Etiology, n (%) |  |  |  | 0.77 |  |
| MetALD | 4 (16.7%) | 3 (18.8%) | 1 (12.5%) |  |  |
| HCV | 8 (33.3%) | 5 (31.3%) | 3 (37.5%) |  |  |
| HBV | 3 (12.5%) | 1 (6.3%) | 2 (25%) |  |  |
| Alcohol | 2 (8.3%) | 1 (6.3%) | 1 (12.5%) |  |  |
| Alcohol and MetALD | 1 (4.2%) | 1 (6.3%) | 0 (0.0%) |  |  |
| Alcohol and Viral | 5 (20.8) | 4 (25%) | 1 (12.5%) |  |  |
| HBV and MetALD | 1 (4.2%) | 1 (6.3%) | 0 (0.0%) |  |  |
| Cirrhosis etiology, n (%) |  |  |  | 1.00 | 0.18 |
| MetALD | 4 (16.7%) | 3 (18.8%) | 1 (12.5%) |  |  |
| Viral | 17 (70.8%) | 11 (68.8%) | 6 (75%) |  |  |
| Alcohol | 3 (12.5%) | 2 (12.5%) | 1 (12.5) |  |  |
| Cirrhosis, n (%) | 16 (66.7%) | 9 (56.3%) | 7 (87.5%) | 0.13 | 0.39 |
| Varicose veins, n (%) | 2 (8.3%) | 1 (6.3%) | 1 (12.5%) | 0.69 | 0.20 |
| Splenomegaly, n (%) | 6 (25.0%) | 4 (25.0%) | 2 (25.0%) | 1.00 | 0.00 |
| Pre-resection elastography, mean (SD) | 9.0±3.6 | 8.2 ±3.2 | 11.1 ±4.2 | 0.13 | 0.78 |
| AFP median (IQR) | 3 (2 - 20) | 3 (3 - 64) | 3.5 (2.0 - 13.0) | 0.40 | -0.60 |
| DAAs, n (%) | 4 (16.7%) | 3 (18.8%) | 1 (12.5%) | 0.70 | 0.17 |
| TACE Treatment, n (%) | 1 (4.2%) | 0 (0.0%) | 1 (12.5%) | 0.33 |  |
| BCLC (A), n (%) | 17 (70.8%) | 11 (68.8%) | 6 (75%) | 0.75 | 0.14 |
| HVPG, mean (SD) | 5.8 ±2.6 | 5.8 ±2.7 | 5.8 ±1.4 | 0.98 | -0.01 |
| Liver resection, n (%) |  |  |  | 0.43 | 0.61 |
| Segmentectomy | 15 (62.5%) | 9 (56.3%) | 6 (75.0%) |  |  |
| Bisegmentectomy | 7 (29.2%) | 6 (37.5%) | 1 (12.5%) |  |  |
| Hepatectomy | 2 (8.3%) | 1 (6.3%) | 1 (12.5%) |  |  |
| Recurrence treatment, n (%)  Laboratory | 9 (90.0%) | 2 (100%) | 7 (87.5%) | 0.60 |  |
| Total bilirubin, mean (SD) | 0.8 ±0.3 | 0.8 ±0.3 | 0.7 ±0.2 | 0.66 | -0.20 |
| Platelets, median (IQR) | 172.500 (150.000 - 213.500) | 173.000 (150.500 - 248.000) | 171.000 (137.500 - 178.000) | 0.56 | -0.41 |
|  |  |  |  |  |  |

**Supplementary Table S2**. List of the antibodies used in the study.

| **Marker** | **Fluorophore** | **Reference** |
| --- | --- | --- |
| CD3 | Pacific Blue | BioLegend, 300431 |
| CD4 | BUV395 | BD Biosciences, 563550 |
|  | AlexaFluor700 | BD Biosciences, 557922 |
| CD8 | Brilliant Violet 605 | BioLegend, 344742 |
| CD19 | Brilliant Violet 711 | BioLegend, 302246 |
|  | PE | eBioscience, 12-0199-42 |
| CD56 | APC | eBioscience, 17-0566-42 |
|  | BUV395 | BD Biosciences, 563554 |
| CD25 | PE | BioLegend, 356134 |
|  | Brilliant Violet 785 | BioLegend, 302638 |
| CD16 | Brilliant Violet 785 | BioLegend, 302046 |
| PD-1 | FITC | BioLegend, 369310 |
| CD69 | PerCP | BioLegend, 310928 |
| CXCR6 | Brilliant Violet 510 | BD Biosciences, 743598 |
| LAG-3 | PE-Cy7 | BioLegend, 369310 |
| CD127 | APC-eFluor780 | eBioscience, 47-1278-42 |
| CD39 | Brilliant Violet 510 | BioLegend, 328219 |
| NKG2D | Alexa Fluor 700 | R&D Systems, FAB139N-100 |
| DNAM-1 | Brilliant Violet 605 | BioLegend, 338323 |
|  | Brilliant Violet 711 | BioLegend, 338334 |
|  | Purified | BD Biosciences, 559786 |
| TIGIT | Brilliant Violet 605 | BioLegend, 372172 |
| CD96 | BB515 | BD Bioscience, 564774 |
|  | Purified | HyCult Biotech, HM2210-100UG |
| Eomes | PE-eFluor 710 | eBioscience, 61-4877-42 |
| T-bet | eFluor660 | eBioscience, 50-5825-80 |
| Viability | VivaFix 353/442 | BioRad, 1351111 |
| IL-10 | eFluor 660 | eBioscience, 50-7108-42 |
| IL-4 | PE | eBioscience, 12-0086-41 |
| IFN-γ | Alexa Fluor 488 | eBioscience, 53-7319-41 |
| TNF-α | Brilliant Violet 785 | BioLegend, 502947 |
| Granzyme B | PE/Cyanine7 | BioLegend, 372213 |
| CD107a | Brilliant Violet 605 | BioLegend, 328633 |
| CD155 | Purified | ThermoFisher, MA5-13493 |
| IgG F(ab’)2 | Purified | ThermoFisher, 31192 |

**Supplementary Table S3.** Immune function markers included in the study of the liver-isolated lymphocytes depending on the tissue source.

| **Marker** | **Biopsy** | **Resection** |
| --- | --- | --- |
| PD-1 | X | X |
| TIM-3 | X | X |
| NKG2D | X | X |
| CD16 | X | X |
| CXCR6 | X | X |
| CD69 | X | X |
| LAG-3 | X | X |
| CD127 | X | X |
| DNAM-1 |  | X |
| CD39 |  | X |
| NKG2D |  | X |
| EOMES |  | X |
| TIGIT |  | X |
| T-BET |  | X |
| CD96 |  | X |
